# Dabigatran-induced Enterococcus Necrotizing Fasciitis in Medically Unfit Patient with Successful Conservative Treatment Outcome: A Case Report

**DOI:** 10.5704/MOJ.2411.012

**Published:** 2024-11

**Authors:** MK Faris, MS Izzuddin, MA Norazrin, MK Thiru

**Affiliations:** Department of Orthopaedic Surgery, Miri Hospital, Miri, Malaysia

**Keywords:** dabigatran, necrotizing fasciitis, enterococcus sp, conservative treatment

## Abstract

A 54-year-old gentleman with underlying hypertension and congestive cardiac disease was diagnosed with left lower limb necrotizing fasciitis following commencement of oral dabigatran. Small bruises on the limb progressed to full-blown sepsis. The calculated perioperative risk for surgery was unfavourable. Immediate diagnosis combined with targeted medical treatment managed to produce a medical wonder.

## Introduction

Dabigatran is a new oral anticoagulant (NOAC) which is prescribed to patients with cardiovascular risk, but with the adverse effect of bleeding tendencies. Necrotizing fasciitis (NF) is a condition that is both limb and life-threatening. Monomicrobial type of NF is second in prevalence with *Enterococcus sp* being a rare causing pathogen. Surgery is inarguably the gold standard of treatment combined with supportive medical optimisation.

Unfortunately, certain medically unfit patients with NF are not amenable for surgery due to adverse calculated perioperative risk, as in our case report.

## Case Report

A 54-year-old gentleman with underlying hypertension and congestive cardiac disease, presented with acute onset of pain and swelling over his left thigh. This coincided with initiation of dabigatran two weeks prior due to diagnosis of left ventricular mural thrombus. Subsequently, he developed small bruises over the thigh which progressively worsened and was associated with fever, chills and rigors.

**Fig. 1:**
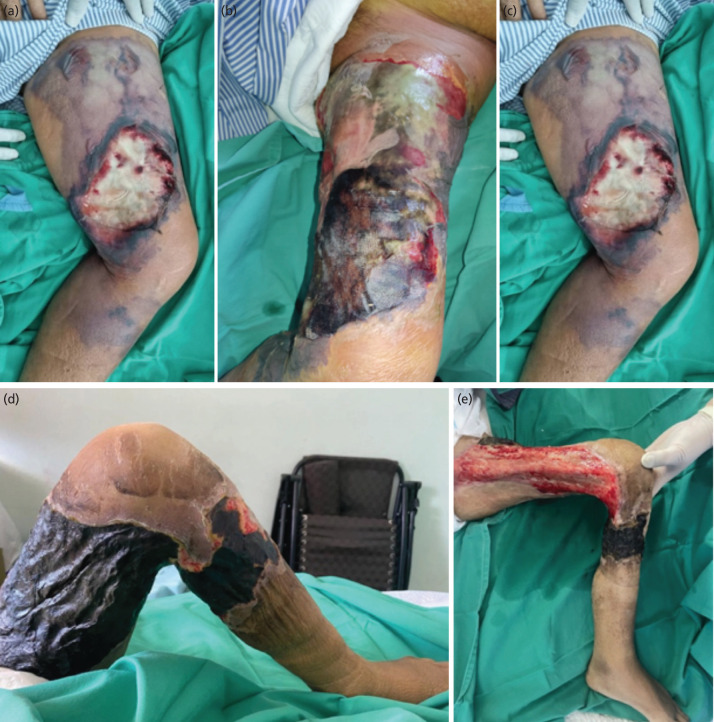
(a) Clinical photograph upon presentation. Large wound over the anteromedial aspect of left thigh, with surrounding skin necrosis and haemorrhagic bullae. Redness, warmness and tenderness with palpable crepitus extend distally to the proximal leg. (b) At day three, the wound progressed with further skin necrosis. (c) At day five, the wound had reduced moisture and the skin necrosis started to demarcate. (d) At two weeks, this wound dried up with eschar coverage. (e) At three months follow-up, partial detachment of the eschar with underlying healthy granulation tissue.

In the emergency department, he was generally unwell with lethargy, jaundice and tachypnoea. There were blackish discolourations with large sloughy wound over his swollen thigh and multiple haemorrhagic bullae extending to the leg (Fig. 1a). He was in shock with coagulopathy and deranged renal and liver functions. A diagnosis of sepsis with left lower limb NF was made.

Following resuscitation, he was stable and required oxygen supplementation, inotropic support and blood product transfusion. Empirical antibiotic was started. He required care in the intensive care unit and was managed via multidisciplinary approach. Detailed investigation was carried out to prepare him for emergent surgery.

Risk stratification for surgery revealed that he was high risk due to coagulopathy, multiorgan dysfunction and poor cardiac reserve with ejection fraction of only 7% and global wall hypokinesia. The Revised Cardiac Risk Index (RCRI) predicted high risk of perioperative major cardiac event.

Family conference was held with the involved teams and they ultimately opted not for surgery in view of high risk. Direction of treatment diverted to optimising medical therapy and rehabilitation.

His condition gradually improved over a two-weeks duration. He was saturating well at room air and not requiring inotropic support. The coagulopathy was normalised and renal function markedly improved. Prophylactic short-acting anticoagulant was reinitiated and subsequently bridged with oral warfarin as a continuance on discharge. The initial empirical antibiotic of piperacillin-tazobactam and clindamycin was changed following repeated blood-culture yields of *Enterococcus sp* to intravenous benzylpenicillin. Total duration of antibiotic was four weeks.

The thigh wound healed but demarcated slowly, Fig. 1b and Fig. 1c, and unfortunately resulted in contractures of hip and knee, Fig. 1d. With ongoing rehabilitation, he was able to mobilise using wheelchair independently. After a month of stay, he was successfully discharged with outpatient reassessment.

On follow-up after three months, he was well, mobilising via wheelchair in his home and semi-dependent for activities of daily living. The necrotic patch dried off with healthy granulation tissue bed underneath, Fig. 1e.

## Discussion

The use of dabigatran as prophylaxis against systemic embolism, stroke and peripheral embolism, has been approved by The Food and Drug Administration (FDA) since October 2010^1^. It is per oral and requires no regular monitoring. Unfortunately, there is a surge of bleeding complications following current outspread usage. The dilemma to surgeons is when patients on anticoagulant require emergent surgery. Various guidelines are available to guide in decision-making citing four considerations; thromboembolic risk, surgical bleeding risk, interruption of anticoagulant and bridging of anticoagulant.

According to the thromboembolism CHA2DS2VASc score, this patient with hypertension and congestive cardiac disease is recommended for anticoagulant. However, it increases his bleeding risk significantly either if he goes for extensive debridement or major limb amputation, namely hip disarticulation, with detrimental consequences. If the decision was to proceed with surgery, he has to undergo anticoagulant withdrawal for the shortest possible duration and ideally restarted within 48-72 hours post-surgery. In this case, the dabigatran would be bridged with short-acting anticoagulant either unfractionated heparin or low-molecular-weight heparin.

The initial bruises were probably a result of intramuscular bleeding and the hematoma acts as a breeding ground for bacteria. Since there was no tissue culture obtained, blood culture result was relied upon. Monomicrobial NF is less common than the polymicrobial type and the commonest associated monomicrobial type are Group-A Streptococcus and Staphylococcus aureus. With the rise of antibiotic resistance, there is increased incidence of gram-negative monomicrobial NF^2^. However, to date, there is no literature comparing the virulence between each monomicrobial infection.

*Enterococcus sp*, a gram-positive coccus previously known as group-D streptococcus, is a rare cause of NF especially monomicrobial. It is a gastrointestinal tract commensal, typically an opportunistic pathogen. It is highlighted by the World Health Organisation (WHO) as rising antibiotic-resistant pathogen. Vancomycin-resistant Enterococci (VRE) causes increase in morbidity and mortality. They do not produce exotoxin like staphylococci and streptococci, but they secrete virulence factors that cause local tissue destruction and induce systemic inflammatory response. The patient’s cardiac condition is negatively affected by the *Enterococcus* septicaemia and subsequently prone to septic shock requiring prolonged inotropic support.

Empirical antibiotic principally should have broad-spectrum activity, target the toxin production and possess good tissue perfusion. Our National Antibiotic Guideline 2019 suggests Piperacillin/Tazobactam and Clindamycin as first-line treatment. Piperacillin/Tazobactam is the preferred broad-spectrum beta-lactam which covers both gram-positive and gram-negative infection while the addition of Clindamycin has good efficacy in reducing toxin production by the Group-A Streptococcus. The initial antibiotic regime should then be tailored to the culture result. In general, *Enterococcus sp* responds well to beta-lactam antibiotics. Therefore, this patient was prescribed high-dose intravenous benzylpenicillin and continued for two weeks.

Surgical debridement or amputation are the mainstay of treatment with the aim of promptly remove the source of infection and this patient ideally required a hip disarticulation. It is performed as the last option where infection is overwhelming due to significant peri and postoperative mortality, both short and long-term^3^. According to The American Society of Anaesthesiologist (ASA) physical status classification, the patient is categorised as ASA-IV which is severe systemic disease that is a constant threat to life^4^ and also, he has high cardiac-risk as per RCRI^5^. This prompted the decision for non-operative treatment.

Despite not being able to perform surgery, medical therapy must be optimised to give the patient the highest chances of survival. Survivability is determined by swift detection, multidisciplinary approach with immediate treatment. Limb deformity and disfigurement is a common sequalae contributed by wound progression and joint contracture, therefore patients must be counselled regarding permanent disability from these complications. The best prevention is by early physical rehabilitation and mobilisation with adequate analgesia. The range-of-motion exercise routine should incorporate both agonist muscle strengthening and antagonist muscle stretching with the goal of getting the joint to the fullest range of stretch and maintain muscle volume. Orthosis may be helpful in some cases.

In conclusion, the widespread use of anticoagulant such as dabigatran increases the incidence of major bleeding complications. This may cause complication of infection especially amongst the immunocompromised and high-risk group. Swift detection and appropriate antibiotic initiation improve survivability. Multidisciplinary approach offers best care for the patient and even though patient is not fit for surgery, medical treatment can be optimised with good outcomes.
